# The Pathology and Molecular Genetics of Sarcomatoid Renal Cell Carcinoma: A Mini-Review

**DOI:** 10.15586/jkcvhl.2017.70

**Published:** 2017-05-22

**Authors:** Shuanzeng Wei, Tahseen Al-Saleem

**Affiliations:** Department of Pathology, Fox Chase Cancer Center, Philadelphia, PA, USA

**Keywords:** carcinosarcoma, pathology reporting, sarcomatoid renal cell carcinoma, TP53, VHL

## Abstract

Sarcomatoid renal cell carcinoma is a highly aggressive tumor. It is not a distinct histologic entity as it can be found in any subtypes of renal cell carcinoma. Recent molecular and genetic evidence suggest that sarcomatoid component is transformed from a common progenitor of the associated renal cell carcinoma, and the TP53 gene plays a pivotal role in this process. The presence of sarcomatoid carcinoma indicates poor prognosis, which also correlates with the amount of the sarcomatoid component. Therefore, the presence and quantity of sarcomatoid component should be reflected in pathology reports. However, pathology reporting seems to vary among laboratories prompting the need for a unified reporting system. We propose a pathology reporting system similar to that of transformed follicular lymphoma that is consistent with the molecular pathogenesis to ensure uniform reporting.

## Introduction

**Figure 1:**
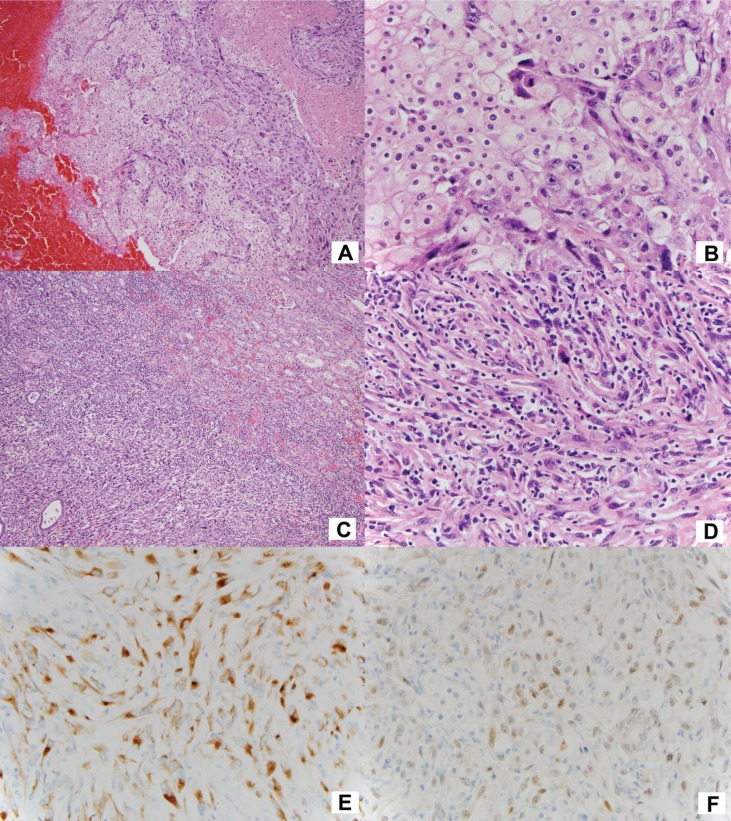
(A) Chromophobe carcinoma mixed with sarcomatoid carcinoma, (B) high power of (A), (C) pure sarcomatoid carcinoma invading the renal parenchyma, (D) high power of the sarcomatoid carcinoma, (E) positive CAM 5.2 (keratin) staining, and (F) positive PAX8 staining.

Renal cell sarcoma used to be the common terminology for all malignant mesenchymal neoplasms of the kidney until Griffith, in 1949, described that many of them also contained renal cell carcinoma (RCC) (1, 2). Carcinosarcoma was then used to better reflect the nature of these tumors. Later, the term sarcomatoid RCC was used by Farrow et al. because they believed these tumors were metaplastic transformation of carcinoma. They regarded it as a separate histologic type to stress the highly aggressive behavior (3). Since sarcomatoid transformation can be present in any RCC subtypes, in current classification schemes, sarcomatoid RCC is not a distinct pathologic subtype of RCC, rather a specific histologic feature (2, 4). Since clear cell RCC is the most common renal carcinoma, 79%–87% of sarcomatoid RCC are associated with this subtype. Chromophobe RCC (Figure 1A and B) accounts for 7%–7.5%, followed by papillary (4%–8%), unclassified (2%–4%), and collecting duct (2%) RCC (5, 6). About 15% of patients who develop stage IV disease have sarcomatoid RCC (7). Sarcomatoid RCC is an independent prognostic factor (8–10) and is associated with death from RCC of all three subtypes: clear cell, papillary, and chromophobe. The presence of sarcomatoid RCC is also significantly associated with poor outcome even in Fuhrman grade IV clear cell RCC (6). However, some investigators found sarcomatoid RCC did not significantly correlate with survival (11, 12).

About 3% of renal carcinomas are purely sarcomatoid (13). The generally accepted explanation is that the sarcomatoid component overgrows and replaces the carcinoma with which it is originally associated (13). Therefore, in practice, thorough gross and histological examination is critical. Although carcinoma component cannot be identified, the malignant spindle cells are generally positive for keratin and PAX8 (Figure 1C–F) (14, 15), which distinguish from bona fide sarcoma of the kidney. Sarcomatoid RCC demonstrates desmosomal cell junctions and poorly formed primitive cell junctions by ultrastructural analysis (16, 17). This intriguing phenomenon of sarcomatoid transformation is not only interesting at a molecular level but also important for patient management with the expanding horizons of targeted therapies. Herein, we briefly review the recent progress in the molecular genetics of sarcomatoid RCC and the potential clinical application. We also propose an easy and reproducible system for reporting the pathology of sarcomatoid RCC.

## Genetic alterations in sarcomatoid RCC

Jones et al., using X-chromosome inactivation analysis, found that both sarcomatoid and carcinomatous components of RCCs are derived from the same progenitor cell (18) suggesting that the sarcomatoid cells may undergo a morphologic transformation from carcinoma. This mechanism is known as epithelial to mesenchymal transition (EMT) (13, 18). In this process, carcinoma cells lose epithelial phenotype and gain mesenchymal feature (19, 20). Recent clinical and preclinical results showed that the disrupted changes leading to EMT can be potential targets of treatment for RCC patients (20). Jiang et al. analyzed 12 cases of sarcomatoid RCC using comparative genomic hybridization technique and found that chromosomal gains are less frequent than chromosomal losses. In the sarcomatoid component of RCC, alteration in chromosomes 4q, 7p21-22, 11q22-23, and 13q were identified (21). Brunelli and colleagues found frequent gains of chromosomes 1, 2, 6, 10, and 17 in sarcomatoid chromophobe RCC in contrast to the loss of these chromosomes in classic chromophobe RCC. These multiple gains of chromosomes were identified in both carcinomatous and sarcomatoid components (22). Our group recently reported that sarcomatoid transformation in RCC is associated with increased chromosomal imbalances with gains of 1q and 8q, losses of 9q, 15q, 18p/q, and 22q compared to non-sarcomatoid RCC (23). Frequently, sarcomatoid RCCs do not have the characteristic chromosome abnormality of the histologic type of RCC it is associated with, which suggests that sarcomatoid component may arise from genetically atypical subclonal tumor cells or from the same progenitor cells (24, 25).

## Molecular alterations

The von Hippel–Lindau (VHL) gene plays a central role in the pathogenesis of clear cell RCC. The important function of pVHL is to target and degrade hypoxia inducible factor (HIF). HIF can activate downstream targets including GLUT1, CA9, and VEGF. Tickoo et al. assessed the expression of HIF in 34 cases of sarcomatoid RCCs. Sarcomatoid RCC associated with clear cell RCC maintained higher HIF pathway expression compared to that associated with non-clear cell RCC (26). Interestingly, Oda et al. found that carcinomatous components had lower TP53 mutation (14%, 2/14) compared to sarcomatoid component (79%, 11/14) (27). Malouf et al. analyzed 26 cases of sarcomatoid RCC using genomic profiling (including 37 introns from 19 commonly rearranged genes and 3230 exons of 236 in carcinoma). They performed genomic profiling of both sarcomatoid and carcinomatous components in three cases, two of which showed identical mutational profiles, and another case harbored commonly disrupted genes. The most frequently involved genes included TP53 (42%), CDKN2A (27%), VHL (35%), and NF2 (19%) (28). Bi et al. studied normal, carcinomatous, and sarcomatoid components of 21 cases of sarcomatoid RCC using exome sequencing. They found that the carcinomatous and sarcomatoid components shared 42% of somatic single nucleotide variants (SSNVs). Sarcomatoid component demonstrated a higher overall SSNV burden and increased recurrent LOH on chromosomes 1p, 17p, 9, 10, 14, 18, and 22. The sarcomatoid and carcinomatous components shared some genes commonly found in clear cell RCC including VHL. They also found biallelic TP53 mutations in 32% of sarcomatoid component; however, there was no TP53 mutations in carcinomatous component. These findings provide solid evidence that both components may arise from a common progenitor or from a tiny subclone that could not be detected by the present level of resolution (23). These discoveries are important for the postoperative management of these tumors as immune modulation or even vaccines may be incorporated as part of adjuvant therapy with the hope of eliminating these precursors and decreasing the chances of recurrence.

## Pathology reporting

As an important prognostic factor, the presence of sarcomatoid RCC on pathologic examination is considered as one of the most important findings by many clinicians; therefore, clear statement of the presence of sarcomatoid component should be an integral part of pathology report (4). The amount of sarcomatoid component varied with a mean of 40%–50% (ranging from 1% to 100%) (5). Shuch et al. found that an increased percentage of sarcomatoid component is associated with a worse prognosis (7). Zhang et al. also reported that the amount of sarcomatoid component is greatly associated with patient’s death from RCC. Each 10% increase in the amount of sarcomatoid RCC was associated with a 6% increased risk of death from RCC. Patients with greater than 30% sarcomatoid component were 52% more likely to die from RCC compared to patients with less than 30% sarcomatoid component (29). The sarcomatoid component includes fibrous, leiomyomatous, rhabdoid, osteoid, or chondroid transformations (5, 30, 31). These uniform patterns of sarcomatoid RCC and the degree of pleomorphism do not affect clinical behavior (2, 6, 30). Coagulative tumor necrosis can be seen in 90% of sarcomatoid RCCs (6). In many cases, the RCC components are of Fuhrman grade III or IV (5, 6). However, by convention, all sarcomatoid RCCs are assigned to Fuhrman grade IV because of the dismal prognosis (5, 6, 15, 32).

Appropriate pathology reporting is an important aspect of cancer care. Shuch et al. reported that tumor classification was omitted in 28% of pathology reports, which may interfere with the selection of systemic therapy and enrollment into adjuvant clinical trials (33). Various terminologies have been and are being used, such as sarcomatoid differentiation, dedifferentiation (28), component, features, and progression. With the progress of our understanding of the molecular/genetic mechanisms driving sarcomatoid RCC, some of these rather popular terms as “differentiation” may be obsolete and even misleading. The inconsistency may cause confusion to the clinician. Since the sarcomatoid carcinoma represents a high-grade tumor, its presence warrants a separate diagnosis with an estimate of the proportion of sarcomatoid component and RCC, similar to the reporting system of diffuse large B cell lymphoma transformation of follicular lymphoma. The diffuse large cell lymphoma and any co-existing follicular lymphoma component are reported separately with their grades and percentages (34, 35).

## Suggestion for a new pathology reporting system

For the above reasons, the reporting of sarcomatoid RCC by pathologists can be slightly modified. Reporting each component separately can reflect the fact that the two components diverge at a stem cell level and facilitate their filing in and retrieval from data bases.

The pathology report for sarcomatoid RCC can read (1) RCC (subtypes, Fuhrman grade, and percentage) and (2) sarcomatoid carcinoma (subtypes, Fuhrman grade IV, and percentage).

We believe that enforcing the reporting in this way reflects the molecular pathogenesis of sarcomatoid RCC and renders reporting of the features and percentage of the sarcomatoid RCC more uniform by pathologists. We hope that reporting the two components separately may also encourage separate molecular studies and search for targeted therapies for either or both components.

## Conclusion

This review highlights that the sarcomatoid component originates from the transformation of the pre-existing RCC, and TP53 gene seems to play a pivotal role in this process. Since the presence of a sarcomatoid component is closely associated with outcome, thorough gross and histological examination is critical. The presence and quantity of sarcomatoid component are important for patient management and, therefore, should be reflected in the pathology report.

## Conflicts of interest

The authors declare no potential conflicts of interest with respect to research, authorship, and/or publication of this article.
